# *SLC39A8* is a risk factor for schizophrenia in Uygur Chinese: a case-control study

**DOI:** 10.1186/s12888-019-2240-2

**Published:** 2019-09-18

**Authors:** Xuemin Jian, Jianhua Chen, Zhiqiang Li, Zhijian Song, Juan Zhou, Wei Xu, Yahui Liu, Jiawei Shen, Yonggang Wang, Qizhong Yi, Yongyong Shi

**Affiliations:** 1 Bio-X Institutes, Key Laboratory for the Genetics of Developmental and Neuropsychiatric Disorders (Ministry of Education) and the Collaborative Innovation Center for Brain Science, Shanghai Jiao Tong University, Shanghai, 200030 People’s Republic of China; 2 Shanghai key laboratory of Sleep Disordered Breathing, Shanghai Sixth People’s Hospital, Shanghai Jiao Tong University, Shanghai, People’s Republic of China; 3 Shanghai Key Laboratory of Psychotic Disorders, Shanghai Mental Health Center, Shanghai Jiao Tong University School of Medicine, Shanghai, 200030 People’s Republic of China; 4 Affiliated Hospital of Qingdao University and Biomedical Sciences Institute of Qingdao University (Qingdao Branch of SJTU Bio-X Institutes), Qingdao University, Qingdao, Shandong 266003 People’s Republic of China; 5 Institute of Social Cognitive and Behavioral Sciences, Shanghai Jiao Tong University, Shanghai, 200030 People’s Republic of China; 6 Institute of Neuropsychiatric Science and Systems Biological Medicine, Shanghai Jiao Tong University, Shanghai, 200030 People’s Republic of China; 7 Department of Neurology, School of Medicine, Renji Hospital, Shanghai Jiao Tong University, Shanghai, 200127 People’s Republic of China; 8 Psychological Medicine Center, The First Affiliated Hospital of Xinjiang Medical University, Urumqi, People’s Republic of China; 9 Shanghai Changning Mental Health Center, Shanghai, 200030 People’s Republic of China; 10 Department of Psychiatry, First Teaching Hospital of Xinjiang Medical University, Urumqi, Xinjiang, 830054 People’s Republic of China

**Keywords:** *SLC39A8*, Schizophrenia, Case-control study, Meta-analysis, Uygur Chinese

## Abstract

**Background:**

Schizophrenia is a severe mental disease with high morbidity and heritability. The *SLC39A8* gene is located in 4q24 and encodes a protein that transports many metal ions. Multiple previous studies found that one of the most pleiotropic single nucleotide polymorphisms (SNPs) in *SLC39A8*, rs13107325, is associated with schizophrenia in the European population. However, the polymorphism of this locus is rare in other populations. In China, the Han Chinese and the Uygur Chinese are two ethnic populations that originate from different races.

**Methods:**

A case-control study was conducted with 983 schizophrenia cases and 1230 healthy controls of the Chinese Uygur population. To validate the most promising SNP, meta-analyses were conducted with the Han Chinese and the European PGC2 data sets reported previously.

**Results:**

A susceptible locus, rs10014145 (*p*_allele_ = 0.014, *p*_allele_ = 0.098 after correction; *p*_genotype_ = 0.004, *p*_genotype_ = 0.032 after correction) was identified in case-control study of the Chinese Uygur population. Further, the association between rs10014145 and schizophrenia was supported by a meta-analysis of Han and Uygur Chinese samples (pooled OR [95% CI] =1.10 [1.03–1.17], Z = 2.73, *p* = 0.006). The association between rs10014145 and schizophrenia was not significant in a meta-analysis of combined Chinese and European samples (pooled OR [95% CI] =1.07 [1.00–1.14], Z = 1.88, and *p* = 0.06). In addition, the “CCAC” haplotype of rs4698844-rs233814-rs13114343-rs151394 was significantly associated with schizophrenia in Uygur Chinese (*P* = 0.003, corrected *p* = 0.012).

**Conclusions:**

The results of this study support that *SLC39A8* is a susceptible gene for schizophrenia in the populations of Han Chinese and Uygur Chinese in China, further studies are suggested to validate the association.

**Electronic supplementary material:**

The online version of this article (10.1186/s12888-019-2240-2) contains supplementary material, which is available to authorized users.

## Background

Schizophrenia is a severe chronic neuropsychiatric disease characterized by hallucinations, delusions and cognitive deficits with high morbidity and heritability [1]. The disease affects up to 1% of the world’s population in a lifetime [2]. Although many pharmacological drugs are used to treat schizophrenia, their efficiency for many patients is poor [3].

The individual heritability of schizophrenia is 60–85% [2]. The etiology of schizophrenia remains far from being fully understood. Multiple genetic studies shed light on the importance of genetic factors. Psychiatric disorders are induced by both numerous genes each of them having a relatively small effect and environmental factors. To date, several susceptibility loci for schizophrenia have been identified by multiple genome-wide association studies (GWASs) [4–11]. The validation of these susceptibility variants of schizophrenia in different human populations remains important, which can provide for a meaningful understanding of its population genetic architecture and may provide more confident and precise targets before pathophysiological or new therapeutic experiments.

The origins and genetic characteristics of different populations are generally different. In China, the Han Chinese population is the main ethnic group and the Chinese Uygur population is one of the minorities. The European population and the Uygur population have a Caucasian origin whereas that of the Han Chinese population is Mongolian. The Uygur Chinese primarily reside in Xinjiang Province, which is located on the northwest border of China, in the middle of Asia. This region experienced unceasing migrations and intermarriages during the Silk Road trade. Additionally, the Uygur population in the region has stable diet habits and lifestyle. These circumstances have all had an important influence on the typical genetic structure formed in the Chinese Uygur population [12, 13]. The genetic structure in this region is proposed to be an admixture of the East and the West [14]. Therefore, the study of the genetic etiology of schizophrenia in this population is important.

Solute carrier family 39 member 8 (*SLC39A8*) belongs to the solute-carrier superfamily (SLC39). The SLC39 family plays an important role in maintaining metal ion homeostasis and is highly conserved across different species. *SLC39A8* encodes a protein named ZIP8, which is responsible for the transport of the essential metals including ferrum (Fe^2+^), manganese (Mn^2+^) and zinc (Zn^2+^), and the nonessential neurotoxic metal cadmium (Cd^2+^) [15, 16]. In 2012, Carrera et al. first uncovered the association between rs13107325 in *SLC39A8* and schizophrenia in a Galician population [17]. Rs13107325 is an exonic single nucleotide polymorphism (SNP) that alters the protein sequence from alanine (Ala; C allele) to threonine (Thr; T allele) at site 391. Thereafter, multiple studies validated the association between rs13107325 and schizophrenia. Based on whole-genome sequencing, rs13107325 is considered as the probably most functional missense mutation in 4q24 which is a region associated with schizophrenia [18]. In 2014, the Psychiatric Genomics Consortium (PGC) identified a new risk SNP around *SLC39A8* for schizophrenia that has strong linkage disequilibrium with rs13107325 [19]. In addition, *SLC39A8* is associated with many different traits such as blood pressure [20, 21], body mass index [22], Crohn’s disease [23], serum levels of manganese [24] and HDL-cholesterol [25] and all the associations are linked to the rs13107325 variant. Thus, for the missense variant rs13107325, *SLC39A8* is one of the most pleiotropic genes involved in many biological processes. However, the T allele of rs13107325 accounts for only 8% in European samples and this polymorphism is almost absent in Asians and Africans according to the 1000 Genome Project phase 3 [18]. Recently, Li et al. performed a GWAS and meta-analysis of Chinese samples (7699 schizophrenia cases and 18,327 controls, called the Bio-X sample) and the PGC2 samples (35,476 schizophrenia cases and 46,839 controls, called the PGC2 sample), and no SNPs in the *SLC39A8* locus was found to be associated with schizophrenia genome-wide significantly (*p* < 5E-8) [11].

Although the significant association between *SLC39A8* and schizophrenia in European Caucasian has been established, this study investigated whether the association between *SLC39A8* and schizophrenia was also present in the Chinese Uygur population, which is considered to be genetically admixed [14]. A case-control association study was conducted to investigate the relationships between seven SNPs in *SLC39A8* and schizophrenia in 983 unrelated schizophrenia cases and 1230 healthy controls of the Chinese Uygur population. To validate the most promising SNP, meta-analyses was conducted with Han Chinese and PGC2 datasets reported previously.

## Materials and methods

### Samples/subjects

A total of 983 unrelated schizophrenia cases (611 males and 372 females) and 1230 healthy controls (635 males and 595 females) were recruited in this study. The mean age ± SD of schizophrenia cases and healthy controls were 40.59 ± 12.35 years and 43.03 ± 13.12 years, respectively. All samples including cases and controls were recruited from the Chinese Uygur population living in Xinjiang Province, China. Most patients were out-patients, but some were stable in-patients. All patients were interviewed by at least two independent psychiatrists strictly according to Diagnostic and Statistical Manual of Mental Disorders Fourth Edition (DSM-IV) based on the Structured Clinical Interview for DSM-IV Axis I Disorders (SCID-I) [26]. The healthy controls were recruited by means of advertisement and announcements on bulletin boards from the general Uygur population. Before collecting blood samples, the healthy controls were screened by psychiatrists. Healthy controls with a family history of psychiatric disorders or with a severe medical illness were excluded. The purpose and potential risks of the study were fully described to the participants and the participants signed informed consents before data collection. The study proposal and procedures were reviewed and approved by the local Ethical Committee of Human Genetics. This study was conducted in accordance with The Code of Ethics of the World Medical Association (Declaration of Helsinki).

### SNP selection, DNA extraction and genotyping

*SLC39A8* gene is located in chromosome 4q24 and has a full length of 83,528 bp DNA. *SLC39A8* contains 8 exons and 7 introns in total.

**Fig. 1:**
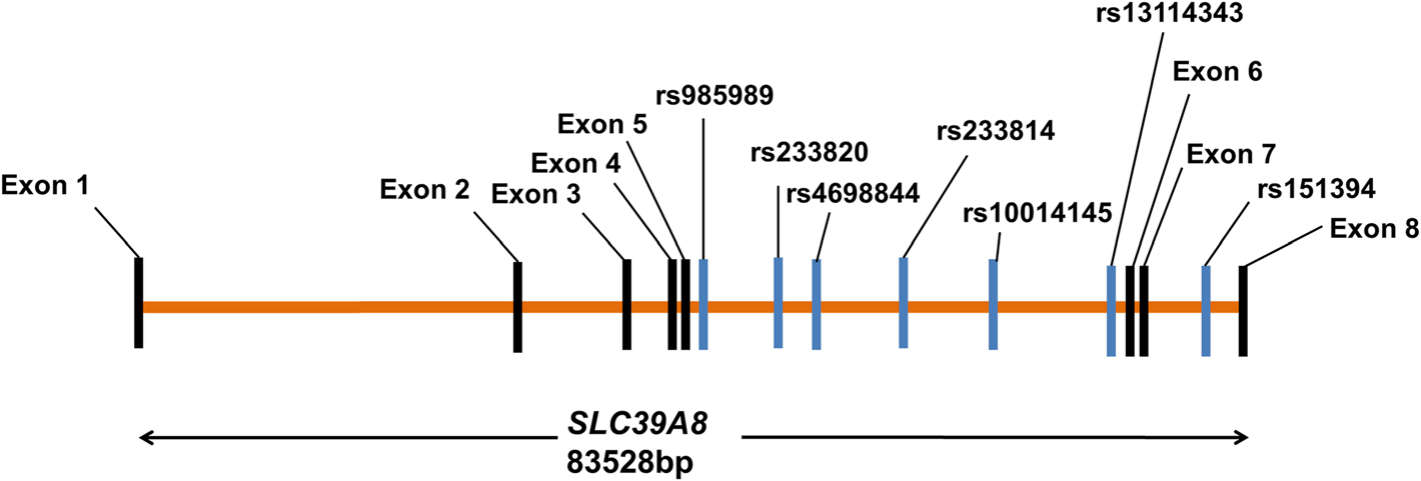
Positions of seven single nucleotide polymorphism in the *SLC39A8* gene

The common SNPs of *SLC39A8* was selected from the HapMap (http:// hapmap.ncbi.nlm.nih.gov/) of the Han Chinese population in Beijing, China. Subsequently, a total of seven tag SNPs including rs233814, rs233820, rs10014145, rs4698844, rs151394, rs985989, and rs13114343 across the *SLC39A8* gene were selected by Haploview software version 4.2. The linkage disequilibrium r^2^ threshold was set as 0.8 and the minor allele frequency was greater than 0.05. All seven SNPs of *SLC39A8* are intron variants. The relative location of these 7 SNPs in *SLC39A8* were designated by Vector NTI (www.invitrogen.com/VectorNTI) and are shown in Fig. 1. Table 1 also provides the information of these seven SNPs.

**Table 1:** Information of the single nucleotide polymorphisms (SNPs) in SLC39A8

SNP ID	Chromosome	Function	Polymorphism	Call rate^a^
rs233814	102,287,546	Intron variant	C/T	0.996
rs233820	102,301,031	Intron variant, UTR variant 3 prime	C/G/A	0.994
rs10014145	102,279,420	Intron variant	A/G	0.97
rs4698844	102,296,823	Intron variant	C/T	0.995
rs151394	102,263,495	Intron variant	C/G	0.987
rs985989	102,304,098	Intron variant	G/T	0.984
rs13114343	102,268,259	Intron variant	A/G	0.99

^a^ Each call rate was calculated in combined cases and controls

The genomic DNA of the subjects was extracted from peripheral blood samples using a Quick Gene DNA whole blood kit L (FUJIFILM) according to the manufacturer’s protocol. The SNPs in this study were genotyped by the Sequenom MassARRAY matrix-assisted laser desorption ionization-time of flight (MALDI-TOF-MS) mass spectrometry platform (Sequenom Inc., San Diego, CA, USA).

### Statistical analyses

For the single site association study, the allele and genotype frequency calculations, the pair-wise linkage disequilibrium analysis were all conducted by the SHEsisPlus online software platform (http://shesisplus.bio-x.cn/SHEsis.html) [27, 28]. All of the *p* values presented are two-sided and only *p* < 0.05 were considered as significant. The *p*-values were adjusted using the Bonferroni correction method. The Bonferroni correction states that if an experimenter is testing n independent hypotheses on a set of data, then the statistical significance level that should be used for each hypothesis separately is 1/n-fold times what it would be if only one hypothesis was tested. Statistical power was post hoc calculated using the G*Power 3.1.9.4 [29].

### Meta-analysis

Association statistics from the Han Chinese Bio-X and European PGC2 datasets were extracted for the most significant SNP in the Chinese Uygur population analysis [11]. The meta-analysis was performed using RevMan5.3. Cochran’s Q test and *I*^*2*^ were used to detect the heterogeneity of the odds ratios. If *P* > 0.05 and *I*^*2*^ < 50%,no significant heterogeneity occurred among the analyses and the fixed effects model (Mantel–Haenszel) was used to pool the ORs; and if *p* < 0.05 or *I*^*2*^ > 50%, random effects model was selected [30].

## Results

The average call rate of all SNPs was 0.988 (Table 1). In the healthy control group, no deviation from Hardy–Weinberg equilibrium was found at the significance threshold being *p* < 0.05(Additional file 1: Table S1).

### Single SNP association analysis

The allele and genotype of seven polymorphic SNPs in *SLC39A8* for both schizophrenia and normal control groups of Uyghur Chinese were statistically analyzed as shown in Table 2. Rs233814, rs10014145, rs4698844, rs151394, rs985989, and rs13114343 had two alleles and rs233820 had three alleles. As shown in Table 2, rs10014145 (*p*_allele_ = 0.014, Chi^2^ = 5.999, OR [95% confident interval (CI)] =1.196 [1.036~1.382]; *p*_genotype_ = 0.004, Chi^2^ = 10.666), showed both allele and genotype were significantly associated with schizophrenia. However, the allele association between rs10014145 and schizophrenia was eliminated after Bonferroni correction (*p*_allele_ = 0.014, *p*_allele_ = 0.098 after correction; *p*_genotype_ = 0.004, *p*_genotype_ = 0.032 after correction).

**Table 2:** Single site association analysis result of seven single-nucleotide polymorphisms in case-control samples of Uygur population

SNP	Allele frequency			OR [95%CI]	Chi^2^	*P*-value	Corrected *p*	Power	Genotypes frequency			Chi^2^	*P* value	Corrected *p*
Rs233814	C	T							C/T	T/T	C/C			
Cases	1276 (0.651)	682 (0.348)		0.968 [0.855~1.096]	0.255	0.613		0.223	420 (0.429)	131 (0.133)	428 (0.437)	2.867	0.249	
Control	1580 (0.643)	872 (0.356)							570 (0.464)	151 (0.123)	505 (0.411)			
Rs233820	C	G	A						C/G	G/G	A/A			
Cases	739 (0.376)	1142 (0.582)	81 (0.041)	NA [NA~NA]	1.09	0.579		NA	429 (0.437)	330 (0.336)	1 (0.001)	1.194	0.956	
									C/A	C/C	G/A			
									26 (0.026)	142 (0.144)	53 (0.054)			
Control	927 (0.38)	1427 (0.583)	86 (0.035)						C/G	G/G	A/A			
									543 (0.445)	413 (0.338)	1 (8.2e-04)			
									C/A	C/C	G/A			
									26 (0.021)	179 (0.146)	58 (0.047)			
Rs10014145	A	G							A/A	G/G	G/A			
Cases	1436 (0.758)	458 (0.241)		1.196 [1.036~1.382]	5.999	0.014*	0.098	0.999	540 (0.57)	51 (0.053)	356 (0.375)	10.666	0.004*	0.032*
Control	1895 (0.789)	505 (0.21)							762 (0.635)	67 (0.055)	371 (0.309)			
Rs4698844	C	T							C/C	C/T	T/T			
Cases	1295 (0.663)	657 (0.336)		0.929 [0.819~1.053]	1.284	0.249		0.663	441 (0.451)	413 (0.423)	122 (0.125)	2.999	0.223	
Control	1586 (0.646)	866 (0.353)							512 (0.417)	562 (0.458)	152 (0.123)			
Rs151394	G	C							G/G	C/G	C/C			
Cases	712 (0.365)	1236 (0.634)		0.946 [0.836~1.071]	0.756	0.384		0.454	143 (0.146)	426 (0.437)	405 (0.415)	3.071	0.215	
Control	917 (0.378)	1507 (0.621)							171 (0.141)	575 (0.474)	466 (0.384)			
Rs985989	C	A							C/A	A/A	C/C			
Cases	1040 (0.533)	910 (0.466)		0.936 [0.831~1.055]	1.146	0.284		0.523	496 (0.508)	207 (0.212)	272 (0.278)	1.308	0.519	
Control	1244 (0.517)	1162 (0.482)							604 (0.502)	279 (0.231)	320 (0.266)			
Rs13114343	G	A							G/G	A/A	G/A			
Cases	1005 (0.514)	949 (0.485)		1.091 [0.968~1.23]	2.08	0.149		0.884	260 (0.266)	232 (0.237)	485 (0.496)	2.179	0.336	
Control	1303 (0.536)	1127 (0.463)							356 (0.293)	268 (0.22)	591 (0.486)			

Abbreviations: *OR* odds ratio, *CI* confidence interval

Significant *P* values were corrected using Bonferroni correction (multiplied by the number of testing SNPs).* *P* values< 0.05

### Haplotype analysis

**Fig. 2:**
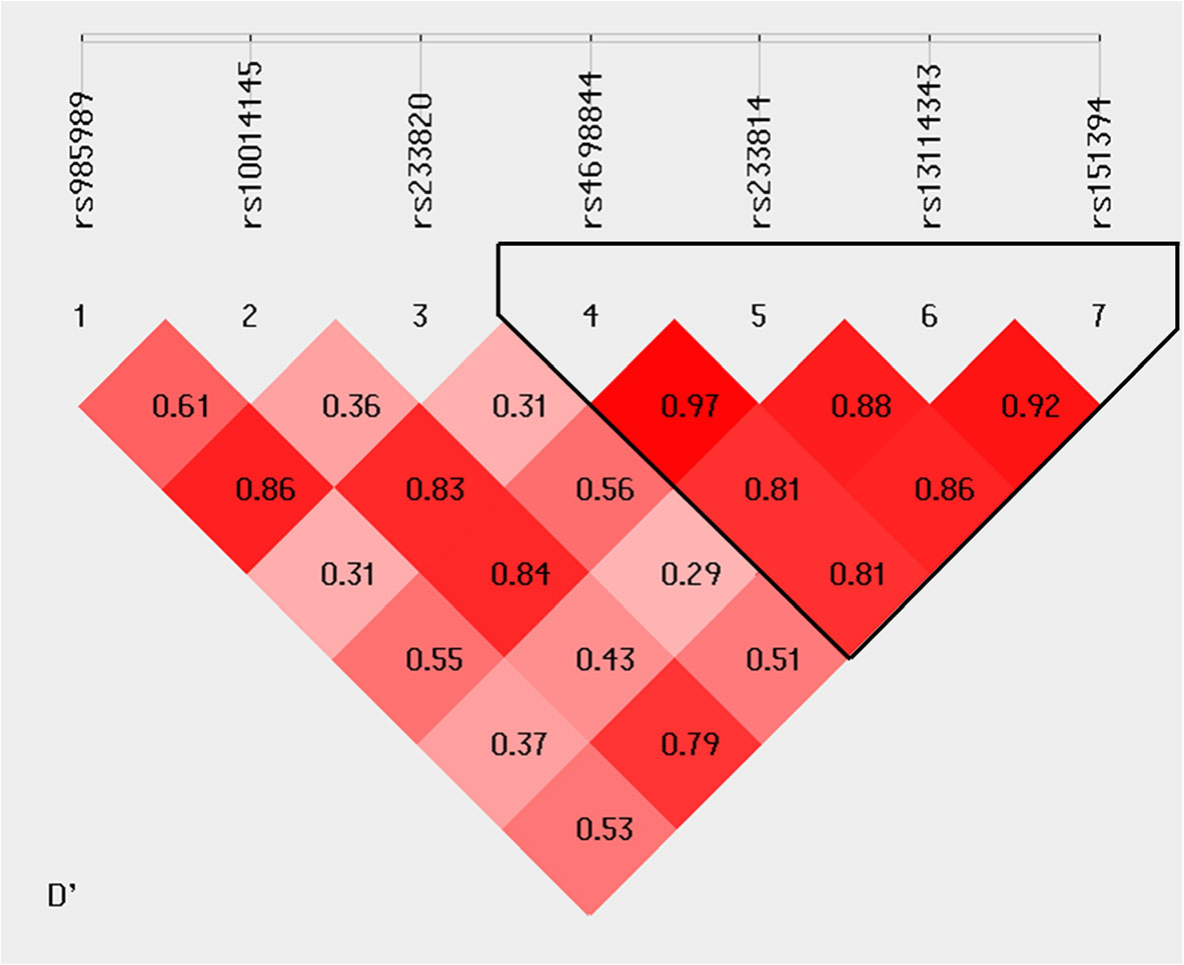
Linkage disequilibrium analysis of seven SNPs in the schizophrenia samples of the Uygur population

The pairwise linkage disequilibrium D’ values among the seven SNPs were calculated in different sample sets. The SNPs with D’ > 0.75 in separated sample sets were classified as in the same block and rs4698844-rs233814-rs13114343-rs151394 was identified as one haplotype block as shown in Fig. 2. In this block, one haplotype “CCAC” showed significant association with schizophrenia (corrected *p* = 0.012, OR [95%CI] =1.296 [1.09~1.54]) (Table 3).

**Table 3:** Haplotype analysis of SLC39A8 in case-control samples of Uygur population

Haplotype	Case (freq)	Control (freq)	OR [95% CI]	Chi^2^	*p*	Corrected *P*	Power
rs4698844-rs233814-rs13114343-rs151394							
CTGG	622 (0.316)	795 (0.323)	0.969 [0.853~1.1]	0.231	0.63		0.177
CCAC	298 (0.151)	298 (0.121)	1.296 [1.09~1.54]	8.688	0.003*	0.012*	0.98
CCGC	271 (0.137)	349 (0.141)	0.967 [0.815~1.147]	0.147	0.701		0.195
TCAC	595 (0.302)	767 (0.311)	0.957 [0.842~1.089]	0.428	0.512		0.301

### Further validation of rs10014145

**Fig. 3:**
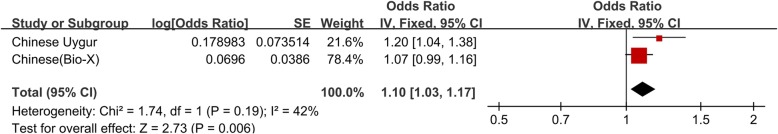
Forest plot of association between rs10014145 and schizophrenia in Chinese samples including Uygur and Han Chinese. OR, odds ratio; CI, confidence interval. Area of the square represents the weight of each statistical sample; horizontal lines represent OR and 95% CI of rs10014145 in two independent samples. The black diamond represents the total 95% CI of rs10014145 estimated in meta-analysis

**Fig. 4:**
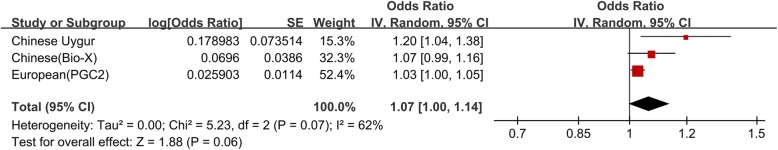
Forest plot of the association between rs10014145 and schizophrenia in Chinese and European populations OR, odds ratio; CI, confidence interval

Meta-analyses of the association between rs10014145 and schizophrenia were conducted. First, a meta-analysis was performed for the Uygur Chinese samples and the Han Chinese (Bio-X) samples. Because no heterogeneity was detected in the combined two independent samples (*I*^2^ = 42%, Chi^2^ = 1.74, *p* = 0.19), the fixed effects model was used to test the significance of rs10014145 in these samples. A significant association was detected between rs10014145 and schizophrenia in the combined Chinese Uygur and Han Chinese populations (pooled OR [95% CI] =1.10 [1.03–1.17] Z = 2.73, and *P* = 0.006) as shown in forest plot of Fig. 3. Furthermore, a meta-analysis was conducted including the Chinese multiple ethnic samples and the Psychiatry Genomics Consortium (PGC2) of European samples. The random effects model was chosen for further analysis because heterogeneity was detected in the three samples (*I*^2^ = 62%, Chi^2^ = 5.23, *p* = 0.07). The forest plot of this meta-analysis of the association between rs10014145 and schizophrenia in the Chinese population and European samples is shown in Fig. 4. The association between rs10014145 and schizophrenia was not validated in three samples (pooled OR [95% CI] =1.07[1.00–1.14], Z = 1.88, *p* = 0.06).

## Discussion

The association between *SLC39A8* and schizophrenia in European populations was discovered in multiple studies, and all researches then focused on the most pleiotropic variant, rs13107325. However, rs13107325 is monomorphic and the T-allele with schizophrenia risk is completely nonexistent in African and Asian populations according to the 1000-Human-Genome project. The explanation of why this polymorphism disappeared in these populations is ascribed to recent positive selection [31–34]. For this report, a case-control study was conducted in the Chinese Uygur population and rs10014145 in *SLC39A8* was identified as significantly associated with schizophrenia in both allele and genotype distributions; however, after correction for multiple testing, the association was only significant for genotype distributions (*p*_allele_ = 0.014, *p*_allele_ = 0.098 after correction; *p*_genotype_ = 0.004, *p*_genotype_ = 0.032 after correction). This association was further validated by the meta-analysis of the multiple Chinese ethnic groups (*P* = 0.006). Although this association was not validated by the meta-analysis of the Chinese population with the European population, the statistical *p*-value was close to 0.05(*p* = 0.06). In conclusion, a new risk locus in Chinese Uygur population was identified in this study, providing new evidence of the association between *SLC39A8* and schizophrenia.

*SLC39A8* encodes a transmembrane protein named ZIP8. ZIP8 transports numerous metal ions such as ferrum, manganese, and zinc from the extracellular environment or intracellular compartments to the cytosol. These metal ions are necessary for many physiological processes in the brain; however, in excess, these ions may cause neurotoxicity [35–37]. For example, the over 20% of zinc distributed in synaptic vesicles of glutamatergic neurons demonstrates the functional relevance of this metal in neurotransmission [38, 39]. In addition, many of these ions can be cofactors of the multiple enzymes that function in the nervous system, such as monoamine oxidase (dopamine), tryptophan hydroxylase (serotonin), tyrosine hydroxylase (catecholamines), glutamine synthetase and superoxide dismutase. Of note, *SLC39A8* can also transport the non-essential, toxic metal cation, cadmium [40]. Cadmium can mimic calcium which plays an important role in neurotransmission; and the exposure to acute doses of cadmium cause immediate damage in the central nervous system and other organs [41]. Zhang, et al. found that the ZIP8 Ala391-to-Thr391 substitution had an effect on intracellular cadmium accumulation and they suggested that pleiotropic effects of rs13107325, which is involved in multiple biological characters including schizophrenia, may attribute to cadmium-induced cell toxicity, highlighting the prominent effect of cadmium transmission [42].

This study identified a new risk locus for schizophrenia, rs10014145. Although rs10014145 is an intronic SNP of *SLC39A8*, it has been reported that rs10014145 is correlated with cadmium concentrations in human blood and urine. Women who carry AG or GG of rs10014145 have a higher concentration of erythrocyte cadmium than those with the AA genotype [43]. In this study, the frequency of genotype GA was significantly higher in patients with schizophrenia than that in normal controls, whereas the frequency of genotype AA was relatively higher in the normal counterparts. The SZDB eQTL data show an association between rs10014145 and three solute-carrier family genes, *SLC16A2, SLC25A23*, and *SLC5A10* (*p* = 0.040, 0.013 and 0.036 respectively). However, no correlation was detected between rs10014145 and *SLC39A8* [44]. Moreover, rs10014145 is predicted to have regulatory potential according to the RegulomeDB analysis [45]. On the basis of the collected results, we propose that the association of *SLC39A8* with schizophrenia partly depends on its function in the transport of metal ions, particularly cadmium transportation and in the maintenance of brain homeostasis.

In addition, gene expression pattern and epigenetic regulation provide additional evidence that *SLC39A8* is relevant to schizophrenia. *SLC39A8* is widely expressed in many tissues, including the brain and is one of the most significantly up-regulated genes in schizophrenia [46]. Furthermore, in the comparison of the post-mortem human brain tissue of schizophrenia patients and healthy controls, *SLC39A8* is one of the most differentially methylated genes in CpG islands of the promoter region, as determined by genome-wide DNA methylation analysis [47]. The aberrant expression pattern of *SLC39A8* may indicate that the gene is involved in the genesis of schizophrenia.

*SLC39A8* is one of 14 members affiliated with the SLC39 family that are responsible for the transport of metal ions. Other family members in the SLC39 family are also identified as relevant to psychiatric disorders. For example, *SLC39A3* is associated with bipolar disorder according to GWAS analyses [48, 49]. *SLC39A11* was revealed as one of the most significant genes in a GWAS of major depressive disorder although it did not reach genome wide significance [50].

## Conclusions

In conclusion, this study provides new evidence of the association between *SLC39A8* and schizophrenia. The relevance of rs13107325 to schizophrenia is widely described; however, this susceptibility locus in the European population is almost monomorphic in other populations. In this study, a case-control study was performed and a new risk genetic locus for schizophrenia, rs10014145, was found in *SLC39A8* in the Chinese Uygur Ethnic population. The association between rs10014145 and schizophrenia was further confirmed through meta-analysis. The primary limitation of this study was the limited sample size. In addition, the genetic structure of schizophrenia in Chinese Uygur population requires further exploration. Although the risk locus of *SLC39A8* for schizophrenia in the Chinese population was validated, further functional studies are necessary to support this association.

## Additional file

Additional file 1 **Table S1** Hardy–Weinberg equilibrium analysis of 7 single-nucleotide polymorphisms in case-control samples of Uygur population (DOCX 16 kb)

## Data Availability

All data generated or analysed during this study are included in this published article.

## Abbreviations

DSM: Diagnostic Criteria of American Diagnostic and Statistical Manual of Mental Disorders
GWAS: Genome-wide association study
LD: Linkage disequilibrium
OR: Odds Ratio
PGC: Psychiatric Genomics Consortium
*SLC39A8*: solute carrier family 39 member 8
SNP: Single-nucleotide polymorphism
UTR: Untranslated region
