# Supplementary material for: SLC39A8 is a risk factor for schizophrenia in Uygur Chinese: a case-control study
Source: BMC Psychiatry. 2019 Sep 18;19:293. doi: 10.1186/s12888-019-2240-2 (PMC6751796; doi:10.1186/s12888-019-2240-2)
Supplement: Supplementary file 1 — Table S1 Hardy–Weinberg equilibrium analysis of 7 single-nucleotide polymorphisms in case-control samples of Uygur population (DOCX 16 kb) [file 12888_2019_2240_MOESM1_ESM.docx]

Additional file 1: Table S1 Hardy–Weinberg equilibrium analysis of 7 single-nucleotide polymorphisms in case-control samples of Uygur population

| SNP | Chi^2^ in case | p in case | p in control |
| --- | --- | --- | --- |
| rs233814 | 2.962 | 0.227 | 0.88 |
| rs233820 | 1.74 | 0.883 | 0.729 |
| rs10014145 | 0.601 | 0.74 | 0.054 |
| rs4698844 | 2.686 | 0.26 | 0.993 |
| rs151394 | 3.167 | 0.205 | 0.956 |
| rs985989 | 0.47 | 0.79 | 0.983 |
| rs13114343 | 0.039 | 0.98 | 0.744 |
